# Detection of Dengue Virus-Specific IgM and IgG Antibodies through Peptide Sequences of Envelope and NS1 Proteins for Serological Identification

**DOI:** 10.1155/2020/1820325

**Published:** 2020-08-04

**Authors:** Pradeep Kumar Nagar, Deepali Savargaonkar, Anupkumar R. Anvikar

**Affiliations:** ICMR–National Institute of Malaria Research, Sec-8, Dwarka, New Delhi 110077, India

## Abstract

Dengue is an acute febrile illness caused by positive-sense single-stranded RNA virus, belonging to the family *Flaviviridae* and genus *Flavivirus*. Transmission of virus among the individuals occurred by blood-feeding *Aedes* mosquitoes. This virus has four serotypes differentiated on the basis of antibody neutralization assay. At present, there is no particular treatment or vaccine candidate available for dengue infection. Approximately 3.9 billion human populations are at risk of dengue virus (DENV) infection. Thus, precise diagnosis of dengue at the early stage is very essential for disease control and effective therapy in order to treat or prevent severe complications. Indeed, the accurate diagnosis of DENV remains a problem because of low detection accuracy along with high testing price. Sensitivity and specificity of available kits vary from test to test, and cross-reactivity with other *Flavivirus* is a challenging issue for diagnosis. In this study, linear epitopes of envelope (E) and NS1 proteins were identified to diagnose the DENV. Whole protein sequences of E and NS1 of DENV were obtained from UniProtKB database. On the basis of algorithm prediction from DNASTAR, BCEPRED, and IEDB data resources, twelve peptides of E (EP1 to EP12) and eight peptides of NS1 (NS1-1 to NS1-8) were selected, which were common in all serotypes. Sequence homologies of peptides with other *Flavivirus* were checked by Multiple Sequence Alignment Tool ClustalX2. Peptide sequences were synthesized chemically by solid-phase peptide synthesis technique. Dengue-specific IgM and IgG (secondary response) antibodies in the patient's antisera were tested with the peptides using ELISA protocol. Peptides EP1, EP2, EP4, EP7, EP10, and EP12 of E protein and NS1-1, NS1-3, NS1-4, NS1-7, and NS1-8 of NS1 protein were considered the best immunoreactive peptides with the sensitivity (73.33-96.66%) and specificity (82.14-100%). Such peptides together can be used to construct the multiple antigen peptides (MAP) or multiplexed microbeads for designing a precise, cost-effective, and easy-to-make peptide-based immunodiagnostic kit for DENV detection.

## 1. Introduction

Dengue virus represents four dissimilar serotypes (DENV1-4) which were classified as *Flaviviridae* family and *Flavivirus* genus [1]. DENV are transmitted to humans by the bite of infected *Aedes* mosquitoes, like most common vector *Aedes aegypti* or to a lesser extent *Aedes albopictus* [2]. The viral genome when entered into the host cell translated directly to a polyprotein complex made up of structural proteins such as nucleocapsid (C), premembrane/membrane (prM/M), envelope (E), and seven nonstructural, viz., NS1, NS2A, NS2B, NS3, NS4A, NS4B, and NS5 proteins [3].

Approximately 390 million dengue infections are estimated annually worldwide [4]. The disease is widespread approximately in 100 countries with more prevalence of cases in Southeast Asia, Americas, and Western Pacific [5]. In India, majority of states are affected by dengue and this is the main cause of hospitalization of people [6]. A few decades earlier, dengue was mainly distributed to urban areas, but now it is common to rural areas as well [7]. Majority of DENV infections are asymptomatic, and approximately 20% of infections showed characteristic dengue fever describe by severe headache, high fever, muscular pain, and body rashes [8, 9]. A minor proportion of dengue cases progresses to its severe forms like dengue hemorrhagic fever (DHF) and dengue shock syndrome (DSS) [10]. DHF and DSS are categorized by higher microvascular permeability, hypovolemia, and petechia [11]. However, diagnosis of diseases at the early stage is very crucial to give an appropriate treatment for the recovery of patients [12, 13].

The E protein displays important function in the protection against DENV because it has the immunodominant epitope sequences that yield virus-neutralizing antibodies [14–16]. This protein contains three different domains: first central domain (EDI) involved in dimerization having fusion peptide (EDII) and EDIII domain has specificity to bind with the surface receptor of host cells [17]. NS1 protein is a glycoprotein (47 kDa) and produced through viral replication, and it is an important antigen to detect infection in the early stage [18, 19]. All *Flavivirus* produced NS1, and it is secreted from infected cells during the early stage of infection. It can be detected within one day after the appearance of primary as well as secondary infection [20]. On the basis of monoclonal or polyclonal antibodies, many types of immunoassays have been commercialized for the detection of DENV NS1 [21, 22]. Serologic methods which are used to detect dengue virus are affected by the cross-reactive antibodies of other *Flavivirus* [23].

Current diagnostic assays identify the virus or nucleic acid through RT-PCR for very early detection and DENV-specific IgM or IgG antibodies through antibody-based test used for after several days of infection [24–28]. Although cross-reactivity of DENV with other *Flavivirus* is a major issue with antibody detection tests [18], the use of native proteins in diagnostic assays would impact not only pricing but also accuracy of result. Hence, the rapid and cheap diagnostic kit with high sensitivity and specificity will be very useful for identification of DENV infection in developing countries. In this study, immunodominant epitopes of E and NS1 proteins of DENV were identified to eliminate the cross-reactivity and to increase the specificity and sensitivity of the assay. Immunoreactivity of synthetic peptides was checked with dengue-specific IgM (early detection) and IgG (secondary infection) antibodies. Eighteen to twenty-five amino acid long peptide sequences were selected on the basis of algorithm prediction from different software programs. After selection, all the peptide sequences were synthesized by Fmoc chemistry. Immunodominant peptide sequences of E and NS1 proteins were identified on the basis of reactivity with the confirmed dengue patient's sera. Peptides showing high immunoreactivity may further be used for the construction of multiple antigen peptides (MAP) to enhance the sensitivity and specificity for the development of the best assay to detect dengue infection.

## 2. Materials and Methods

### 2.1. Clinical Samples

In this study, we include 150 dengue-positive samples which were collected from the Fever Clinic situated at ICMR–National Institute of Malaria Research (NIMR), New Delhi. Samples were collected in the first six days of postsymptom onset and were considered acute-phase samples and convalescent-phase samples after the acute-phase sample. Twenty-eight healthy volunteer samples were used as the negative control. Approval of the study was taken from the Institutional Ethics Committee, ICMR-NIMR, New Delhi, and all the patients were enrolled by receiving their written informed consent.

### 2.2. Peptide Sequences

Full-length sequences of envelope (E) and NS1 proteins were obtained from UniProtKB database. On the basis of different algorithms such as hydrophilicity, antigenicity index, secondary structure, surface probability, and flexibility prediction from the Immune Epitope Database and Analysis Resource (IEDB), DNASTAR, and BCEPRED (crdd.osdd.net/raghava/bcepred) software programs, sequences of peptides (12 of E protein and 8 of NS1 protein) were selected. Envelop (accession no. D6MQ78) and NS1 (accession no. Q06371) proteins of DENV-2 were used as basic sequence for peptide selection. Peptide sequences which were more similar in all the four serotypes (DENV1-DENV4) of dengue virus were selected. All the peptide sequence homology was cross-checked with the JEV, ZIKV, YFV, and chikungunya virus by Multiple Sequence Alignment Tool ClustalX2. Peptides which did not show similarities with other *Flavivirus* were selected for synthesis. Structural localization of peptide sequences was identified by using UCSF Chimera 1.12. All the peptides were synthesized chemically by Fmoc chemistry using solid-phase peptide synthesis (SPPS) technique. Completed peptide sequence was cleaved by trifluoroacetic acid (TFA) from the resin support using free radical scavengers like anisole and thioanisole. Further peptides were purified by gel filtration chromatography and later by HPLC using C18 column (Table 1).

### 2.3. Optimization of ELISA Assay

Different concentration (25 ng, 50 ng, 75 ng, 100 ng, and 200 ng) of peptides of E and NS1 were coated into the ELISA plates. All the peptides were dissolved in 100 *μ*l of 0.1 M carbonate-bicarbonate coating buffer (pH 9.6) and incubated overnight at 4°C. Plates were washed with PBS Tween-20 buffer solution (pH 7.4), 5% BSA solution was used for blocking, and plates were kept at 37°C temperature for 2 hrs. After washing, 100 *μ*l of pooled sera of 25 dengue patients (1 : 100, 1 : 200, and 1 : 400 dilutions) was added and again incubated for 2 hrs at 37°C. Afterward, wells were washed and 100 *μ*l of HRP-conjugated secondary antibody (1 : 1000 dilutions, goat antihuman IgM *μ* chain HRP, Abcam, USA) was added. After this, one-hour incubation was done at 37°C and plates were washed; finally, the color was developed using TMB as a substrate solution and reaction was stopped by using 8N H_2_SO_4_. Absorbance of the developed color was measured at 492 nm. Here, the optimized amount of coating peptides was 200 ng and antisera dilutions were 1 : 200.

### 2.4. Measurement of IgM and IgG Antibodies

An optimized concentration of 200 ng/100 *μ*l of peptides (E and NS1) was coated on a microplate with coating buffer, and ELISA plates were incubated overnight at 4°C. After washing three times by PBS Tween-20 solution and blocking with 5% BSA protein solution, again plates were kept for 2 hrs period at 37°C. 100 *μ*l of the dengue patient's antisera and healthy individuals' (1 : 200 dilutions) was put into duplicate wells of washed plates. 100 *μ*l of goat antihuman IgM-HRP or goat antihuman IgG-HRP conjugate (1 : 1000 dilutions) was added and incubated up to 1 hr at 37°C. Finally, the color was developed same as the above procedure. Samples of healthy volunteers were used as the negative control, and virus lysate of DENV-2 serotype (The Native Antigen Company, UK) was used as a positive control. The patients' sera which showed absorbance equal or higher than the mean value of negative antisera + 2SD (standard deviation) were included as positive.

### 2.5. Diagnostic Performance of ELISA

The sensitivity of peptides was calculated as the [no.of samples (dengue‐infected group) which showed absorbance equal to or greater than (mean + 2SD) of the healthy group divided by the total number of used positive samples] × 100. The specificity was calculated as [no.of samples which showed the absorbance less than mean + 2SD value of the healthy group divided by the total number of samples from the healthy group] × 100.

### 2.6. Statistical Analysis

The data were analyzed using GraphPad Prism 5.0 software. All the values were considered significant which showed *p* value less than 0.05. Results were expressed as mean absorbance + SD with the range.

## 3. Results

### 3.1. IgM (Pooled Antisera) Reactivity with Peptides

**Figure 1:**
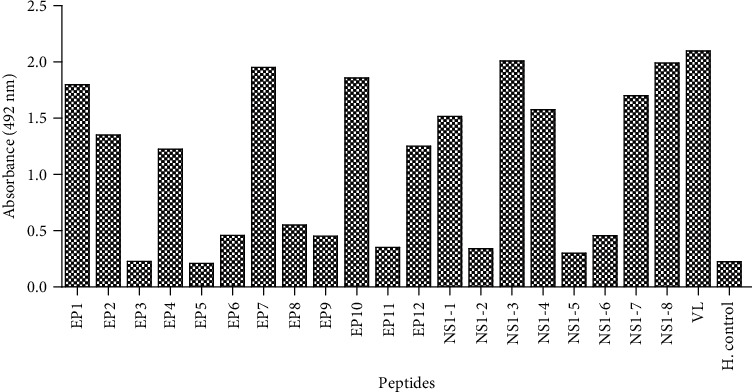
IgM reactivity from the pooled sera of DENV (*n* = 25) patients with synthetic peptides of E and NS1 proteins and virus lysate (VL, positive control) of dengue virus. Reactivity was compared with pooled sera of healthy individuals (*n* = 10) (negative control).

Some patients may respond to one epitope to another, but all may or may not to respond to all epitopes. Thus, we use a pool of sera, to increase the likelihood for detecting a response. Here, few IgM epitopes of envelope E and NS1 proteins were identified. EP1, EP2, EP4, EP7, EP10, and EP12 of E protein and NS1-1, NS1-3, NS1-4, NS1-7, and NS1-8 of NS1 protein showed significant immunoreactivity with dengue-specific IgM antibodies in the patient's sera. These results conclude that specific antibodies against the synthesized peptides are present in the pooled sera. The ELISA results of pooled sera of the dengue cases (*n* = 25) are shown in Figure 1. The rest of the peptides did not show significant reactivity; hence, these peptides have no specific antibodies in the antiserum and are not considered for further immunoreactivity with the individual patient's antisera.

### 3.2. Peptide Reactivity with IgM Antibodies of the Individual Patient's Sera

**Figure 2:**
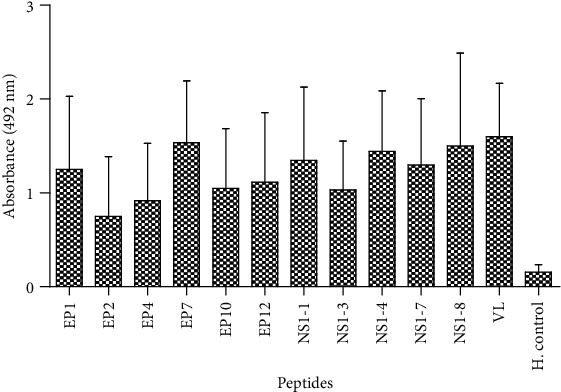
IgM antibody reactivity with six peptides of envelope (E), five peptides of NS1 protein, and virus lysate (VL, positive control) in DENV-positive sera (*n* = 150) and healthy controls (*n* = 28). All the peptides showed *p* < 0.0001, and EP2 showed *p* < 0.001 value.

Further immunoscreening of synthesized peptides of E and NS1 proteins was tested individually with one hundred fifty samples of the dengue patient's sera to know the reactivity pattern of antibodies. Seroreactivity was also checked in 28 healthy individual sera. All peptides which showed OD (optical reading) value equal or higher in comparison to the mean OD value of healthy individuals plus 2SD were concluded as positive. All the immunodominant peptides showed a specific pattern of immunoreactivity with the patient's sera (Figure 2). Peptides which showed sensitivity equal to 70% or higher were confirmed as antigenic in nature. Dengue virus-2 lysate was used as a positive control. Peptides EP1, EP2, EP4, EP7, EP10, and EP12 of E protein and NS1-1, NS1-3, NS1-4, NS1-7, and NS1-8 of NS1 protein showed significant reactivity compared to healthy individuals. EP1, EP7, NS1-4, and NS-8 peptides showed the highest reactivity compared to EP2, EP4, EP12, NS1-1, NS1-3, and NS1-7 with IgM antibodies. EP1 and NS1-8 were identified as the most dominating peptides with a sensitivity of 96.66%, followed by NS1-1, NS1-4, and EP2 with a sensitivity of 95.33%, 94.66%, and 94%, respectively (Table 2).

### 3.3. Peptide Reactivity with IgG Antibodies of the Individual Patient's Sera

**Figure 3:**
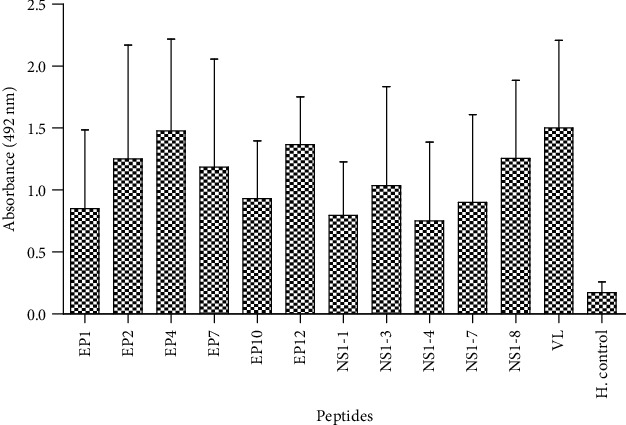
IgG antibody reactivity with six peptides of envelope (E), five peptides of NS1 protein, and virus lysate (VL, positive control) in DENV-positive sera (*n* = 150) and healthy controls (*n* = 28). All the peptides showed *p* < 0.0001 value, and NS1-4 showed *p* < 0.001 value.

The IgG antibody level is very crucial in secondary infection of dengue virus. The peptides which showed immunoreactivity with IgM antibodies were also evaluated to test IgG reactivity. All the immunodominant peptides of E and NS1 proteins showed significant seroreactivity with dengue-specific IgG antibodies (Figure 3). Recognition patterns of IgM and IgG antibodies showed a correlation in reactivity with peptides. Peptides which showed IgM reactivity also showed IgG antibody reactivity which means the same epitopes showed reactivity with IgM and IgG antibodies. Peptides showed sensitivity 73.33 to 94.66% and specificity 82.14 to 100 (Table 2).

### 3.4. Localization of Peptide Sequence in Proteins

**Figure 4:**
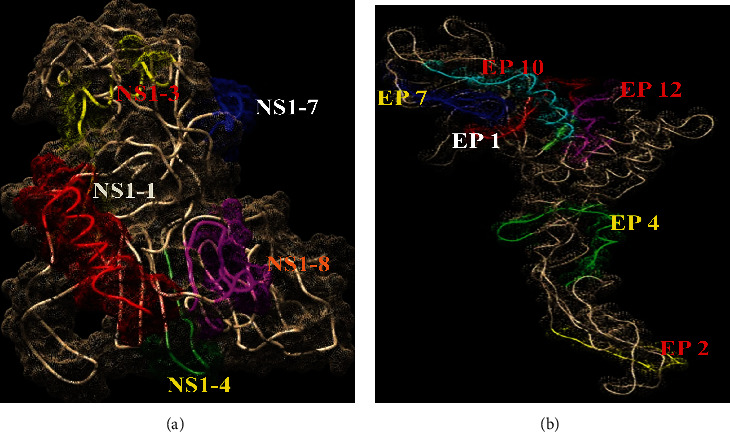
Surface localization of peptides of NS1 (a) and E (b) proteins. All the immunoreactive peptides were present on the surface of proteins. Visualization of peptides was done by using UCSF Chimera 1.12 software.

To check the localization of the peptides on the proteins, UCSF Chimera 1.12 was used. The model of E protein was retrieved from RCSB PDB (Id:4UIF, Chain A of E), and the predicted models of NS1 protein were prepared by I-TASSER (Zhanglab.ccmb.med.umich.edu/ITASSER/output/S552700). Visualization of all the selected peptides in proteins was done using UCSF Chimera 1.12 software. All the immunoreactive peptide sequences were found to be on the surface of proteins as shown in Figure 4. Generally, an immune B cell prefers surface-localized epitopes due to their hydrophilic nature.

## 4. Discussion

In the current situation, no specific therapy is available for dengue. An early and accurate detection of virus plays an important role for better clinical management or to avoid lethal complications. Commercial methods of dengue diagnosis have limitations, including cross-reactivity, high cost, and poor detection accuracy. In this study, we used synthetic peptide-based approach for the detection of dengue-specific IgM and IgG antibodies for diagnosis of dengue virus. Here, we selected the different peptide sequences based on algorithm prediction and screened these peptides with the confirmed dengue patient's sera. Different types of laboratory diagnostic techniques are being used such as isolation of virus, viral RNA, and virus-specific antibody detection [29]. However, poor sensitivity, specificity, and cross-reactivity of existing assays are a big issue. Few studies showed that the sensitivities of the Pan-E dengue and PLATELIA™ dengue NS1 ELISA kits vary with the DENV serotype [30], and the detection level was poor for DENV3 serotypes [31]. PLATELIA kits showed more sensitivity (66%) compared with Pan-E (52%) in confirmed dengue samples, and sensitivity also varied by geographic region [31]. To evaluate the cross-reactivity of diagnostic kits, Guzman et al. showed that Panbio and Focus ELISA kits were highly cross-reactive with other viruses [32]. Finally, sensitivity and specificity of kits, their cost, and cross-reactivity with other *Flavivirus* are the main problems in the current situation. Thus, there is a need to develop a cheap, rapid, and highly sensitive assay for the diagnosis. Most available diagnostic kits of dengue are made on the basis of native structural or nonstructural proteins; here, peptide-based approach can be a good option to complete the above criteria. A study reported to use the peptide analog of immunogenic peptides and raised antisera in rabbit. The antisera of immunized rabbit showed immunoreactivity with the synthetic peptide analog. This proves that peptides can induce antibody response [33]. Also, the peptide-based approach has been tried for the development of a diagnostic assay against many diseases [34]. A study showed that synthetic peptides derived from immunogenic proteins can be the used for developing a diagnostic assay for tuberculosis [35]. Morey et al. showed that diagnosis of chikungunya virus using peptides can be an effective and a more accessible approach [36]. In this study, we focused on structural envelope (E) and nonstructural NS1 proteins. Both proteins are known to be the major targets for diagnosis and have been used in earlier studies [37]. The NS1 protein is the best target for diagnosis of DENV because it presents in several forms: it is secreted from infected cells, has high-level circulation in the blood of patients, and can be detected from the onset of symptoms of diseases. It is the most targeted antigen for dengue diagnosis. Here, we used a combined approach using E and NS1 for IgM and IgG antibody-based diagnosis.

NS1 detection in secondary infection of dengue has limitations due to rapid and amnestic rise in cross-reactive antibodies for NS1 at the acute phase. Due to this, NS1 is sequestered as an immune complex that hinders the detection of NS1 in capture assays. Thus, the kinetics of detection of native NS1 during secondary infections is shorter than that during primary infections. Hence, we selected both structural E and nonstructural NS1 proteins due to the fact that their immune response to one protein may disappear sometimes. Immunodominant peptides were identified by reactivity with confirmed dengue-positive sera. Overall, few sequences EP1, EP2, EP4, EP7, EP10, and EP12 of E protein and NS1-1, NS1-3, NS1-4, NS1-7, and NS1-8 of NS1 protein showed significant reactivity. These peptides also showed significant sensitivity and specificity. All immunoreactive peptides were present on the surface of the E and NS1 proteins confirmed by UCSF Chimera 1.12 software. These peptides showed high immunoreactivity with IgM and IgG antibodies; it seems that E and NS1 proteins induce long-term antibody response in DENV infection. On the basis of the IgM/IgG antibody ratio, primary and secondary dengue infections can be differentiated. Dengue infection will be defined as primary if the IgM/IgG OD ratio is greater than 1.2 (using the patient's sera at 1/100 dilution). The infection is secondary if the ratio is less than 1.2 [38]. We propose that these peptides can be used for the construction of multiple antigen peptides (MAP) [39] or can be attached to multiplexed microbeads [40] for developing a better immunodiagnostic kit for dengue in early and late phases of the disease.

## 5. Conclusion

This study identifies the specific linear peptide sequence of envelope and NS1 proteins, which showed a desired immunoreactivity with the dengue-specific IgM and IgG antibodies in the patient's sera. Peptides EP1, EP2, EP4, EP7, EP10, and EP12 of E protein and NS1-1, NS1-3, NS1-4, NS1-7, and NS1-8 of NS1 were found as the best immunodominant peptides with significant immunoreactivity, sensitivity, and specificity. These peptides fulfill the criteria toward diagnosis of dengue virus. By using another approach like multiple antigen peptides (MAP) or multiplexed microbeads, these peptides can further be used for the development of a diagnostic kit to detect dengue virus.

## Figures and Tables

**Table 1:** 

Peptide sequences of envelope (E) protein:		Seq. no.
EP1	RCIGISNRDFVEGVSGGSWVDIVL	2-25
EP2	NTTTASRCPTQGEP	67-80
EP3	KPTLDFELIKTEA	38-50
EP4	MENKAWLVHRQWFLDLPLPWLPGADT	201-226
EP5	CSPRTGLDFNEMVLLQ	185-200
EP6	VCKHSMVDRGWGNGCGLFGKG	91-111
EP7	KEIAETQHGTIVIRVQYEGDG	310-330
EP8	VVLGSQEGAMHTALTGATEI	251-270
EP9	NIEAEPPFGDSYIIIG	366-381
EP10	SWFKKGSSIGQMFETTMRGA	390-409
EP11	LVLVGVVTLYLGVMVQA	479-495
EP12	RMAILGDTAWDFGSLGGV	411-428
Peptide sequences of NS1 protein:		Seq. no.
NS1-1	MNSRSTSLSVSQVLVGIVTLYLGV	1-24
NS1-2	IKGIMQVGKRSLRPQPTELRY	121-141
NS1-3	TEQYKFQPESPSKLASAIQKA	57-77
NS1-4	ALNDTWKIEKASF	233-245
NS1-5	CHWPKSHTLWSN	251-262
NS1-6	DCGNRGPSLRTTTAS	318-332
NS1-7	PETAECPNTNRAW	166-178
NS1-8	VLESEMVIPKNFAGPKSQ	264-281

**Table 2:** Sensitivity and specificity of peptides. Peptides showed 73.33 to 96.66% of sensitivity and significant range of specificity with the dengue virus-specific IgM and IgG antibodies.

Peptides	IgM antibodies		IgG antibodies	
	True positive/false negative sample (*n* = 150)	Sensitivity/specificity (%)	True positive/false negative sample (*n* = 150)	Sensitivity/specificity (%)
EP1	145/5	96.66/96.43	140/10	93.33/100
EP2	141/9	94/92.85	110/40	73.33/92.85
EP4	140/10	93.33/89.28	135/15	90/85.71
EP7	129/21	86/82.14	128/22	85.33/89.28
EP10	122/28	81.33/92.85	136/14	90.66/92.85
EP12	139/11	92.66/96.43	142/8	94.66/89.28
NS1-1	143/7	95.33/96.43	136/14	90.66/96.43
NS1-3	139/11	92.66/92.85	141/9	94/82.14
NS1-4	142/8	94.66/100	122/28	81.33/100
NS1-7	132/18	88/96.43	133/17	88.66/96.43
NS1-8	145/5	96.66/96.43	131/19	87.33/96.43

## Data Availability

Here, we did not use any type of specific available data in the support of study.
